# Preclinical Assessment of Bacteriophage Therapy against Experimental *Acinetobacter baumannii* Lung Infection

**DOI:** 10.3390/v14010033

**Published:** 2021-12-24

**Authors:** Sandra-Maria Wienhold, Markus C. Brack, Geraldine Nouailles, Gopinath Krishnamoorthy, Imke H. E. Korf, Claudius Seitz, Sarah Wienecke, Kristina Dietert, Corinne Gurtner, Olivia Kershaw, Achim D. Gruber, Anton Ross, Holger Ziehr, Manfred Rohde, Jens Neudecker, Jasmin Lienau, Norbert Suttorp, Stefan Hippenstiel, Andreas C. Hocke, Christine Rohde, Martin Witzenrath

**Affiliations:** 1 Division of Pulmonary Inflammation, Charité–Universitätsmedizin Berlin, Corporate Member of Freie Universität Berlin and Humboldt-Universität zu Berlin, 10115 Berlin, Germany; sandra.wienhold@charite.de (S.-M.W.); markus.brack@charite.de (M.C.B.); geraldine.nouailles@charite.de (G.N.); gopinath.krishnamoorthy@charite.de (G.K.); jasmin.lienau@charite.de (J.L.); andreas.hocke@charite.de (A.C.H.); 2 Department of Infectious Diseases and Respiratory Medicine, Charité–Universitätsmedizin Berlin, Corporate Member of Freie Universität Berlin and Humboldt-Universität zu Berlin, 10115 Berlin, Germany; norbert.suttorp@charite.de (N.S.); stefan.hippenstiel@charite.de (S.H.); 3 Department of Microorganisms, Leibniz Institute DSMZGerman Collection of Microorganisms and Cell Cultures, 38124 Braunschweig, Germany; imke.korf@item.fraunhofer.de (I.H.E.K.); christine.rohde@dsmz.de (C.R.); 4 Department of Pharmaceutical Biotechnology, Fraunhofer Institute for Toxicology and Experimental Medicine (ITEM), 38124 Braunschweig, Germany; claudius.seitz@item.fraunhofer.de (C.S.); sarah.wienecke@item.fraunhofer.de (S.W.); anton.ross@item.fraunhofer.de (A.R.); holger.ziehr@item.fraunhofer.de (H.Z.); 5 Department of Veterinary Pathology, Freie Universität Berlin, 14163 Berlin, Germany; kristina.dietert@fu-berlin.de (K.D.); corinne.gurtner@vetsuisse.unibe.ch (C.G.); olivia.kershaw@fu-berlin.de (O.K.); achim.gruber@fu-berlin.de (A.D.G.); 6 Veterinary Centre for Resistance Research, Freie Universität Berlin, 14163 Berlin, Germany; 7 Central Facility for Microscopy, Helmholtz-Centre for Infection Research (HZI), 38124 Braunschweig, Germany; manfred.rohde@helmholtz-hzi.de; 8 Department of General, Visceral, Vascular and Thoracic Surgery, Charité–Universitätsmedizin Berlin, Corporate Member of Freie Universität Berlin and Humboldt-Universität zu Berlin, 10115 Berlin, Germany; jens.neudecker@charite.de; 9 German Center for Lung Research (DZL), Partner Site Charité, 10117 Berlin, Germany

**Keywords:** bacteriophage, antibiotic resistance, *Acinetobacter baumannii*, pneumonia, preclinical development

## Abstract

Respiratory infections caused by multidrug-resistant *Acinetobacter baumannii* are difficult to treat and associated with high mortality among critically ill hospitalized patients. Bacteriophages (phages) eliminate pathogens with high host specificity and efficacy. However, the lack of appropriate preclinical experimental models hampers the progress of clinical development of phages as therapeutic agents. Therefore, we tested the efficacy of a purified lytic phage, vB_AbaM_Acibel004, against multidrug-resistant *A. baumannii* clinical isolate RUH 2037 infection in immunocompetent mice and a human lung tissue model. Sham- and *A. baumannii*-infected mice received a single-dose of phage or buffer via intratracheal aerosolization. Group-specific differences in bacterial burden, immune and clinical responses were compared. Phage-treated mice not only recovered faster from infection-associated hypothermia but also had lower pulmonary bacterial burden, lower lung permeability, and cytokine release. Histopathological examination revealed less inflammation with unaffected inflammatory cellular recruitment. No phage-specific adverse events were noted. Additionally, the bactericidal effect of the purified phage on *A. baumannii* was confirmed after single-dose treatment in an ex vivo human lung infection model. Taken together, our data suggest that the investigated phage has significant potential to treat multidrug-resistant *A. baumannii* infections and further support the development of appropriate methods for preclinical evaluation of antibacterial efficacy of phages.

## 1. Introduction

Infections caused by multidrug-resistant (MDR) bacteria represent a “slow-motion pandemic” that has been spreading for decades and poses an increasing threat to global health [1]. A recent antimicrobial surveillance program revealed that, with the exception of *Enterococcus* *faecium*, other pathogens such as *Staphylococcus aureus*, *Klebsiella pneumoniae*, *Acinetobacter baumannii*, *Pseudomonas aeruginosa* and Enterobacter species (ESKAPE) were predominantly isolated from patients with bacterial pneumonia admitted to a network of sentinel hospitals in North America, Europe, and elsewhere [2]. Of particular concern is the emergence of *A. baumannii*, a gram-negative opportunistic pathogen that causes severe pneumonia, meningitis, and sepsis and exhibits intrinsic resistance to multiple classes of anti-infectives [3]. Due to its high persistence capabilities, it can thrive under different environmental conditions, including hospital-settings and readily causes outbreaks in intensive care units [4]. Patients on mechanical ventilation are at high risk for infection with *A. baumannii* and resulting pneumonia that is associated with a particularly high mortality rate [5,6]. Adequate therapy for those infections is extremely challenging due to the menacing ability of *A. baumannii* to tolerate treatment or even acquire new resistance mechanisms under ongoing therapy [3,7]. In addition, the remaining drugs that are effective against these bacteria are often hazardous for patients, e.g., nephrotoxic colistin [8]. Given the severity of *A. baumannii* infections and the current challenge to eradicate MDR strains, the implementation of new therapeutic strategies is of high medical value [7]. Accordingly, the World Health Organization recently announced drug-resistant *A. baumannii* as one of the bacteria with critical priority for research and development of new drugs, which are safer and highly potent [9]. Despite the strong impetus globally to develop new drugs, this has proven to be enormously challenging. Alternative anti-infective approaches such as bacteriophage (phage) therapy hold promise owing to its ability to infect and eventually lyse bacteria including MDR strains [10]. Experimental studies [11,12] and patient case reports [13,14] demonstrated the antibacterial potential of phages. Yet, these successful outcomes have been replicated only partially in human clinical trials [15,16,17], which might be attributed to critical gaps in the preclinical development process of phage-based medicine. Evidence-based preclinical data on treatment of lung infections are largely lacking. Especially the knowledge on the choice of infection models, appropriate therapeutic dose and application route, levels of purity, and bioavailability of phages required to effectively treat lung infections, is incomplete [18]. In addition, the development and use of animal models, and their application to reliably assess the efficacy of phage therapy needs more effort. Therefore, preclinical assessments in a model where phages interact with bacteria in the host immunity milieu are required to accurately predict the therapeutic success of phage candidates in clinics [19]. Furthermore, practice-based approaches in which an *A. baumannii* patient isolate is treated with previously isolated and well-characterized phages to avoid time-consuming de novo search, have not been considered regularly. Thus, we tested a clinical MDR *A. baumannii* isolate of a former outbreak [20] for susceptibility against a panel of previously isolated and characterized bacteriophages. After choosing one suitable phage candidate [21], we employed a sequential process to reduce endotoxin levels in the prepared phage suspension. Subsequently, we aimed to evaluate its therapeutic use against *A. baumannii* lung infection in immunocompetent mice and human lungs regarding efficacy and possible adverse effects. Our data show that the chosen phage candidate is effective and does not provoke detectable adverse effects after intratracheal aerosolization in mice.

## 2. Materials and Methods

### 2.1. Ethics

All animal experiments were performed according to ethical guidelines and under permission of institutional (Charité–Universitätsmedizin Berlin) and governmental authorities (LAGeSo, Berlin) and complied with the Federation of European Laboratory Animal Science Association (FELASA) guidelines and recommendations for the care and use of laboratory animals. Human lung tissue samples were freshly obtained from patients undergoing lung resection mainly due to bronchial carcinoma. The study was approved in accordance with ethical guidelines and under permission of the ethical committee of Charité–Universitätsmedizin Berlin (EA2/079/13) and written informed consent was obtained from all patients.

### 2.2. Bacterial Strain and Growth Conditions

For all experiments, *A. baumannii* isolate RUH 2037, kindly provided by Alex F. de Vos and Tom van der Poll was used [20,22]. Drug susceptibility profile was determined with disk diffusion method according to DIN (German Institute for Standardization) 58940 guidelines [23] and resistance to more than two classes of antibiotics, including penicillins, cephalosporins, and quinolones was confirmed (Supplementary Materials Table S1, full susceptibility profile is available online [24]). Bacteria were grown from cryopreserved stocks on Columbia agar containing 5% sheep blood (BD, Heidelberg, Germany) and incubated overnight at 37 °C with 5% CO_2_. Subsequently, single colonies were transferred to trypticase soy broth (TSB, Caso Bouillon, Carl Roth, Karlsruhe, Germany) and incubated at 37 °C and 220 rpm until mid-logarithmic phase (OD_600 nm_ ≈ 0.5). After centrifugation, the pellet was resuspended in sterile Dulbecco’s Phosphate-Buffered Saline (DPBS without Magnesium and Calcium, Thermo Fisher Scientific, Waltham, MA, USA) and adjusted to desired inoculation dose for in vivo or ex vivo experiments.

### 2.3. Production of Phages

After process development, the well-characterized phage vB_AbaM_Acibel004 (family: *Myoviridae*), kindly provided by Maya Merabishvili [21], was manufactured by Fraunhofer ITEM according to quality requirements for a pharmaceutical grade active ingredient [25]. The process sequence included *inter alia* bacterial cell growth of RUH 2037, infection with phages, microfiltration for removal of cell debris, ultra- and diafiltration of phage lysates, one ion exchange chromatographic step for impurity removal and a final microfiltration for bioburden reduction. Two batches were manufactured and adjusted with saline-magnesium (SM) buffer (0.1 M NaCI, 8 mM MgSO_4_, 50 mM Tris-HCI, pH 7.2–7.5) to a concentration of approximately 5 × 10^6^ plaque-forming units (PFU)/mL and 4000 endotoxin units (EU)/mL for in vivo experiments and approximately 5 × 10^9^ PFU/mL and 8000 EU/mL used for ex vivo experiments. Endotoxin concentration was measured using the Chromo-LAL assay (Associates of Cape Cod, East Falmouth, MA, USA). To control for different levels of endotoxins, a mechanically disrupted RUH 2037 lysate was added to SM-buffer at defined concentrations and used as control solution in all experiments.

### 2.4. Negative Staining of Phage vB_AbaM_Acibel004 with Uranyl Acetate for Transmission Electron Microscopy

Thin carbon support films were prepared by sublimation of a carbon thread onto a freshly cleaved mica surface. Phages were adsorbed onto the carbon film, washed first in Tris-EDTA (TE) buffer (10 mM TRIS, 1 mM EDTA, pH 7.0) and secondly in distilled water and then negatively stained with 2% (*w*/*v*) aqueous uranyl acetate, pH 5.0, according to the method of Valentine et al. [26]. Samples were examined in a TEM 910 transmission electron microscope (Carl Zeiss AG, Oberkochen, Germany) at an acceleration voltage of 80 kV and at calibrated magnifications (using a line replica). Images were recorded digitally with a Slow-Scan CCD-Camera (ProScan, 1024 × 1024, Scheuring, Germany) with ITEM-Software (Olympus Soft Imaging Solutions, Münster, Germany). Contrast and brightness were adjusted with Adobe Photoshop 6.0 (Adobe Inc., San Jose, CA, USA).

### 2.5. Field Emission Scanning Electron Microscopy

Samples were fixed with 5% formaldehyde and 2% glutaraldehyde in HEPES buffer (0.1 M HEPES, 0.01 M CaCl_2_, 0.01 M MgCl_2_, 0.09 M sucrose, pH 6.9) for 1 h at 7 °C and washed with TE buffer (20 mM TRIS, 1 mM EDTA, pH 7.0). Glass cover slips with a diameter of 12 mm were coated with a poly-L-lysine solution (Sigma-Aldrich, Taufkirchen, Germany) for 10 min, washed in distilled water and air-dried. 30 µL of the fixed samples were then placed on a cover slip and allowed to settle for 10 min. Cover slips were finally fixed in 1% glutaraldehyde in HEPES buffer for 5 min at room temperature and subsequently washed with TE-buffer before dehydrating in a graded series of acetone (10%, 30%, 50%, 70%, 90%, 100%) on ice for 15 min for each step. Samples in the 100% acetone step were allowed to reach room temperature before another change in 100% acetone. Samples were then subjected to critical point drying with liquid CO_2_ (CPD 300, Leica Biosystems, Wetzlar, Germany). Dried samples were covered with a gold/palladium film by sputter coating (SCD 500, Balzers Union, Balzers, Liechtenstein) before examination in a field emission scanning electron microscope Zeiss Merlin (Carl Zeiss AG, Oberkochen, Germany) using the Everhart Thornley SE detector and the inlens detector in a 75:25 ratio at an acceleration voltage of 5 kV and at calibrated magnifications. Contrast and brightness were adjusted with Adobe Photoshop 6.0. Phage staining and electron microscopy were performed at HZI Braunschweig.

### 2.6. Murine Pneumonia Model

Specific-pathogen-free, female, 8–10 weeks old C57BL/6N mice (Charles River, Sulzfeld, Germany) were kept in randomly assigned groups in individually ventilated cages with 12/12-h light/dark cycles and free access to food and water. To induce *A. baumannii* pneumonia in mice, a prior described method was used with some modifications [22]. Briefly, animals were anesthetized with ketamine/xylazine (80/25 mg/kg body weight intraperitoneally, i.p.) and transnasally infected with 5 × 10^8^ CFU of *A. baumannii* or 20 µL DPBS (sham). Mice were monitored and clinical parameters including body weight and temperature (BAT-12 Microprobe, Physitemp, Clifton, NJ, USA) were measured twice a day. A modified clinical disease score (Supplementary Materials Table S2) based on specific mouse pneumonia signs was assessed prior to euthanasia as described [27,28]. Mice reaching predefined humane endpoints were euthanized.

### 2.7. Murine Phage Therapy and Efficacy Analysis

Twelve h after bacterial or sham infection, a single application of 25 µL phages (final concentration of approximately 5 × 10^6^ PFU/mL) or control solution (buffer) was administered by intratracheal aerosolization following orotracheal intubation under isoflurane anesthesia as described [29], using a microsprayer for rodents (Microsprayer™ application system, Penn-Century Inc. Industries, Ltd., Philadelphia, PA, USA) [30]. At predefined endpoints, mice were anesthetized (ketamine/xylazine 160/75 mg/kg i.p.), tracheotomized, and ventilated. After intracardial heparinization and exsanguination, lungs were perfused with sterile 0.9% NaCl. Bronchoalveolar lavage (BAL) was performed twice using 800 µL DPBS with protease inhibitor (completeMini with EDTA; Roche Diagnostics GmbH, Mannheim, Germany) and processed for cell isolation and CFU quantification. Lungs were divided equally, minced, and digested for isolation of immune cells for flow cytometry analysis or homogenized for analysis of bacterial count. Further, bacterial load was measured in BAL, homogenized spleen (in 1 mL DPBS), and blood (with EDTA as an anticoagulant). Serial dilutions were plated on blood agar plates (5% sheep blood), incubated overnight prior to CFU counting and bacterial load per organ (lung, spleen) and per mL (BAL) was calculated.

### 2.8. Leukocyte Differentiation in BAL, Lungs and Blood

Leukocytes in BAL and lung homogenate were analyzed by flow cytometry (BD FACSCantoTM II, BD, Heidelberg, Germany) as described [27]. In brief, lung homogenate was digested in RPMI media containing collagenase II (Biochrome, Berlin, Germany) and DNAse I (PanReac AppliChem, Darmstadt, Germany) for 30 min at 37 °C and single cell suspension was prepared using 70 µm cell strainer (BD, Heidelberg, Germany). BAL and lung cells were blocked with anti-CD16/CD32 (2.4G2, BD, Heidelberg, Germany) and stained with anti-CD11c (N418, ATCC, Manassas, VA, USA), anti-CD11b (M1/70, eBioscience, San Diego, CA, USA), anti-F4/80 (BM8, eBioscience, San Diego, CA, USA), anti-Ly6G (1A8, BD, Heidelberg, Germany) and anti-Ly6C (AL-21, BD, Heidelberg, Germany), anti-CD45 (30F11, BD, Heidelberg, Germany), anti-Siglec-F (E50-2440, BD, Heidelberg, Germany), and anti-MHCII (M5/114.15.2, eBioscience, San Diego, CA, USA) monoclonal antibodies (mAbs). For calculation of total cell numbers, CountBright Absolute Counting Beads (Thermo Fisher Scientific, Waltham, MA, USA) were used. For gating strategy and staining details refer to Supplementary Materials Figure S1. Blood cell counts were determined in EDTA blood using a cell coulter for veterinarian use (scil Vet abc™, Scil animal care company GmbH, Viernheim, Germany). Remaining blood was centrifuged, and plasma and BAL supernatant (BAL fluid, BALF) were filtered in tubes (Ultrafree CL centrifugal devices, Merck KGaA, Darmstadt, Germany) and stored at −80 °C until further analysis.

### 2.9. Alveolar-Capillary Barrier Permeability Index

To quantify pulmonary vascular leakage, mouse serum albumin (MSA) levels in BAL fluid and plasma were measured by ELISA (mouse albumin ELISA Quantification Set, BETHYL Laboratories, Montgomery, TX, USA) according to manufacturer’s instructions and the BAL/plasma MSA ratio was calculated as the permeability index of the alveolar-capillary barrier as described [27].

### 2.10. Cytokine Quantification

Concentrations of different cytokines and chemokines were measured in BAL fluid and plasma using multiplex assay (Luminex^®^ ProcartaPlex Custom Mix & Match, Affymetrix eBioscience, San Diego, CA, USA) according to manufacturer’s instructions.

### 2.11. Histopathology Analysis

Separate groups were assigned for histopathological examination. Mice were euthanized at indicated time points. Organs were carefully removed, immersion-fixed in 4% buffered formaldehyde solution pH 7.0 (Neolab, Heidelberg, Germany) for 24 to 48 h, embedded in paraffin, cut into 2 μm sections, and stained with hematoxylin and eosin (HE) after dewaxing in xylene and rehydration in decreasing ethanol concentrations as described previously [31]. Histopathological evaluation was performed by board-certified veterinary pathologists blinded to the study groups as described [32].

### 2.12. Immunohistochemistry

For immunohistochemical detection of *A. baumannii*, antigen retrieval was performed using microwave heating (600 W) in 10 mM citric acid (750 mL, pH 6.0) for 12 min. Lung sections were then incubated with a rabbit antibody polyclonal to *A. baumannii* (1:100, kindly provided by José Ramos-Vivas [33]) at 4 °C overnight. Incubation with a random rabbit antibody at the same dilution served as negative control. Subsequently, slides were incubated with a secondary, alkaline phosphatase conjugated goat anti-rabbit (1:500, AP-1000, Vector Labs, Burlingame, CA, USA) antibody for 30 min at room temperature. The alkaline chromogen triamino-tritolyl-methanechloride (Neufuchsin) was used as phosphatase substrate for color development. All slides were counterstained with hematoxylin, dehydrated through graded ethanol, cleared in xylene and coverslipped. Slides were analyzed and images taken using a BX41 microscope (Olympus, Hamburg, Germany) with a DP80 Microscope Digital Camera and the cellSens Imaging Software, Version 1.18 (Olympus, Hamburg, Germany). For overviews, slides were automatically digitized using the Aperio CS2 slide scanner (Leica Biosystems Imaging Inc., Vista, CA, USA) followed by image file generation using the Image Scope Software (Leica Biosystems Imaging Inc., Vista, CA, USA) as described [31].

### 2.13. Phage Efficacy in Human Lung Tissue

Human lung tissue was freshly obtained from tumor-free surgery specimens. Lungs were processed according to published protocols with minor changes [34]. In brief, tissue was cut, weighed, transferred to RPMI medium (Thermo Fisher Scientific, Waltham, MA, USA) in 12-well plates (Falcon^®^ Tissue Culture Plate, Corning, Corning, NY, USA) and incubated at 37 °C with 5% CO_2_ overnight. Following overnight incubation lung tissue was inoculated with 1 × 10^3^ CFU/mL of *A. baumannii*. Thirty min after stimulation, either 100 µL of approximately 5 × 10^9^ PFU/mL phages or endotoxin enriched control solution (buffer) were injected. At defined time points, lung samples were homogenized in 1 mL DPBS and bacterial load determined as described [35]. In brief, serial dilutions were plated on blood agar plates, incubated overnight prior to CFU counting and bacterial load per g lung tissue was calculated. Tissue culture supernatant was collected, filtered, and stored at −80 °C until further analysis. Cytokines in supernatant were measured via ELISA (IL-1β, Invitrogen, Waltham, MA, USA; IL-8, BD, Heidelberg, Germany) and total cytokine production per g lung tissue and medium volume (2.2 mL) was calculated.

### 2.14. Statistical Analysis

Data are presented as box plots depicting median, quartiles and range excluding outliers (open circles), as curves or single values with mean ± SD and analyzed using GraphPad Prism 9 (GraphPad Software Inc., San Diego, CA, USA). CFU data were logarithmized (Y = log(CFU+1)). Cytokine values below lower limit of detection (LLOD) were calculated as LLOD/√2 as described by Croghan et al. [36]. Grouped analyses were performed using 2-way ANOVA and Tukey’s multiple comparisons test. Two groups were compared using 2-tailed, unpaired *t*-test or Mann-Whitney U Test. *p* < 0.05 was considered statistically significant.

## 3. Results

### 3.1. A Single Intratracheal Phage Application Mitigated the Severity of Murine Pneumonia Caused by Drug-Resistant A. baumannii Strain

**Figure 1:**
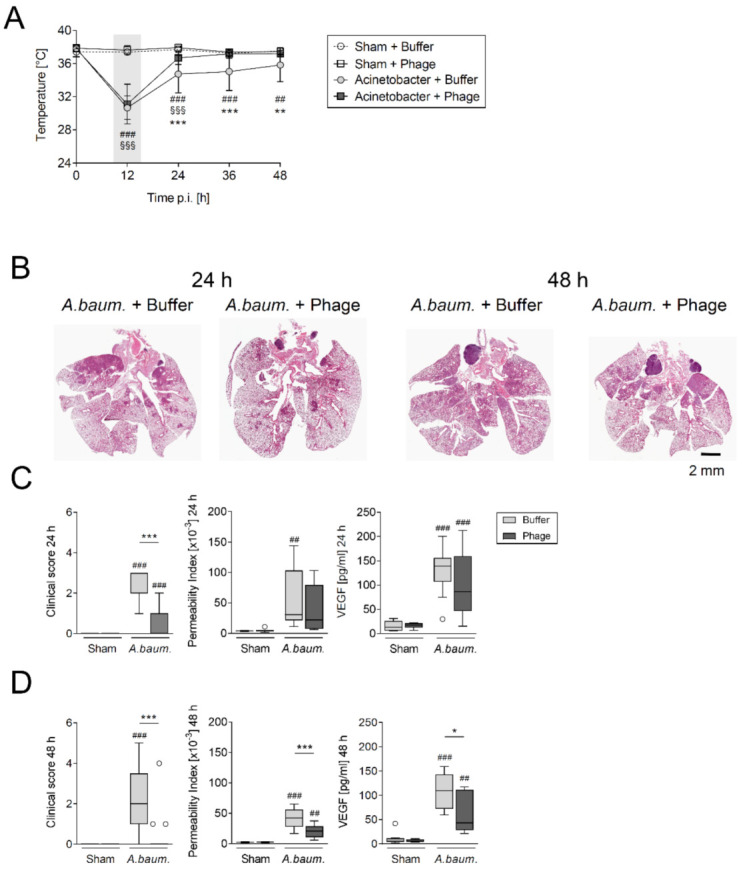
Phage therapy mitigated murine *A. baumannii* pneumonia. Mice were transnasally infected with 5 × 10^8^ CFU *A. baumannii* RUH 2037 or sham-infected (DPBS) and intratracheally treated with specific phage, vB_AbaM_Acibel004, or control solution (buffer) 12 h post infection (p.i.). At defined time points mice were sacrificed for BAL and blood sampling. Separate groups were sacrificed for histological analyses. (**A**) Body temperature curves of all mice subjected to analysis at 24 h (*n* = 11–12; sham-infected groups or *n* = 16–17; *A. baumannii* infected groups) and 48 h (*n* = 12; sham-infected groups or *n* = 17; *A. baumannii* infected groups) are shown combined (mean ± SD). Representative images of H&E-stained whole lung sections from *A. baumannii* infected buffer or phage-treated mice are shown at 24 h and 48 h p.i. (**B**); *n* = 3–4 (sham-infected groups), *n* = 6 (*A. baumannii* infected groups). Murine clinical disease score, permeability index and VEGF levels in BAL were assessed at 24 h (**C**) and 48 h p.i. (**D**). Data are presented as box plots depicting median, quartiles, and range excluding outliers (open circles) and analyzed using 2-way ANOVA and Tukey’s multiple comparisons test. C and D (clinical score), *n* = 11–12 (sham-infected groups) or *n* = 16–17 (*A. baumannii* infected groups; including 3 mice euthanized due to humane endpoints); C and D (permeability index and VEGF levels), *n* = 8 (sham-infected groups) or *n* = 8–11 (*A. baumannii* infected groups). Grey box in A marks time point of phage application. §/#/* indicate significant differences between groups at corresponding time point; § sham + phages vs. Acinetobacter + phages (A), # infected vs. corresponding sham group, *Acinetobacter + buffer vs. Acinetobacter + phages, * *p* < 0.05, ##/** *p* < 0.01 and ###/***/§§§ *p* < 0.001.

Two independent batches of vB_AbaM_Acibel004 phage suspension were produced, and its virulence and host-specificity were confirmed prior testing in animals. Morphology and structural integrity of the prepared phage suspension was verified through electron microscopy analysis (Supplementary Materials Figure S2). Immunocompetent mice were intranasally challenged with 5 × 10^8^ CFU of *A. baumannii* RUH 2037 and developed clinically apparent pneumonia within 12 h characterized by a concomitant decline in body temperature (Figure 1A). Both sham and *A. baumannii* infected mice were subjected to intratracheal aerosolization with either buffer (control group) or phage suspension containing 5 × 10^6^ PFU/mL (phage-treated group). While the buffer-treated *A. baumannii* infected group had a lower body temperature until the end of the experiment (i.e., up to 48 h p.i.), normal body temperature was restored after phage treatment. Additionally, *A. baumannii*-infected mice exhibited significant weight loss. A marginally higher loss of body weight was noted in phage-treated mice only at 24 h p.i. when compared with the buffer-treated group (Supplementary Materials Figure S3). In contrast, no changes in body temperature or body weight were recorded in sham-infected animals regardless of treatment. Histopathological analysis of H&E-stained *A. baumannii* infected lungs revealed widely expansive bronchopneumonia (Figure 1B) with numerous infiltrating polymorphonuclear leukocytes (PMN) causing suppurative inflammation with alveolar edema and necrosis, as previously described [32]. Although the histopathologically determined total cell count remained unaltered by phage treatment (data not shown), spreading of inflammation to the periphery along bronchi and large vessels was less pronounced in phage-treated groups than in buffer-treated animals 24 h p.i. Severity and expansion of lesions were more pronounced 48 h p.i. and notably less prominent in lungs from phage-treated mice with less peripherally disseminated inflammation (Figure 1B). Clinical signs of murine pneumonia, regarding behavior and fur appearance, were milder after 12 h of phage treatment (Figure 1C,D).

Vascular endothelial growth factor (VEGF) among others promotes destabilization of cell-cell connections *[37]* and therefore contributes to development of pulmonary vascular leakage, represented by alveolar-capillary barrier permeability index *[27]*. Accordingly, alveolar VEGF-levels were found elevated in all infected mice (Figure 1C,D). However, there were lower pulmonary vascular leakage index and VEGF levels as well as improvement of clinical signs at 48 h p.i. in phage-treated animals (Figure 1D).

**Figure 2:**
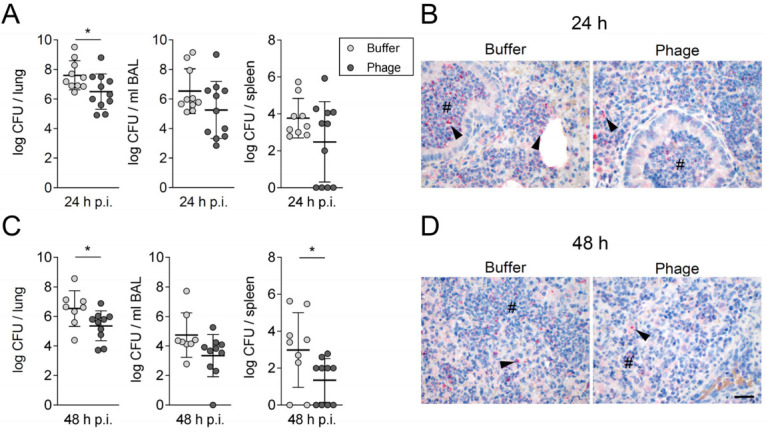
Single intratracheal phage application reduced bacterial burden in mice. Colony forming units (CFU) of *A. baumannii* were quantified in BAL, homogenized half lungs and spleen at 24 h (**A**) and 48 h (**C**) post infection (p.i.). Representative images of immunohistochemically stained lung tissue sections to visualize *A. baumannii* at 24 h (**B**) and 48 h p.i. (**D**). Data are expressed as single values with mean ± SD. CFU data were logarithmized (Y = log(CFU+1)) and tested with unpaired student’s *t*-test; *n* = 9–11 (24 h p.i.); *n* = 8–10 (48 h p.i.) for each group. * *p* < 0.05 between indicated groups; *n* = 3–4 (sham-infected groups), *n* = 6 (*A. baumannii* infected groups) per time point for histology. #: neutrophils, arrowheads: bacteria, scale bar 50 μm.

Consistent with the improved clinical condition of the infected mice, the bacterial burden in lungs was reduced in phage-treated animals by approximately 1 log10 at 24 h p.i. (Figure 2A). This was confirmed by the lower immunohistochemical detection of *A. baumannii* in lungs of phage-treated animals (Figure 2B). At 48 h p.i., phage-treatment was associated with lower bacterial counts in lungs and spleen, whereas bacterial counts in BAL remained unaltered (Figure 2C,D).

### 3.2. Phage Therapy Did Not Additionally Increase Lung Inflammation in Mice

Potential immune reactions due to locally acting or systemic dissemination of phage particles or components of phage-lysed bacteria remain a concern for therapeutic usage of phages. To test whether the presence of phages in vivo could stimulate a proinflammatory response, we analyzed changes in immune cell populations and inflammatory mediators in both sham and *A. baumannii* infected immunocompetent mice treated with phages or buffer.

**Figure 3:**
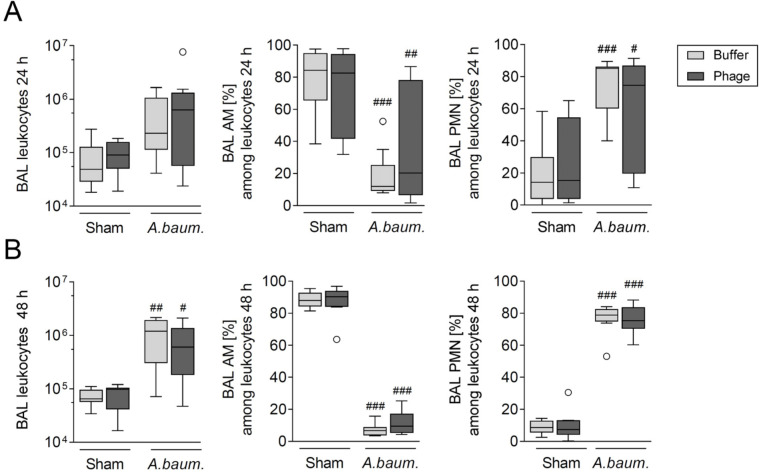
Phage therapy did not increase lung inflammation in mice. Numbers of leukocytes and percentages of alveolar macrophages (AM) and polymorphonuclear leukocytes (PMN) in BAL of mice were analyzed at 24 h (**A**) and 48 h p.i. (**B**) by flow cytometry. Data are presented as box plots depicting median, quartiles and range excluding outliers (open circles) and analyzed using 2-way ANOVA and Tukey´s multiple comparisons test; *n* = 8 (sham-infected groups); *n* = 8–11 (*A. baumannii* infected groups). # indicates significant difference between infected vs. corresponding sham group. # *p* < 0.05, ## *p* < 0.01 and ### *p* < 0.001.

As anticipated, *A. baumannii* infection resulted in mobilization of innate immune cells from vascular spaces to the site of infection. Influx of neutrophils into alveolar spaces was evident in terms of total numbers and proportional increase at 24 h (Figure 3A, Supplementary Materials Figures S4A and S5A) up to 48 h p.i. (Figure 3B, Supplementary Materials Figures S4B and S5B). At 24 h p.i., relative numbers of blood neutrophils had more than doubled, at the expense of relative lymphocyte numbers (Supplementary Materials Figure S6A). In response to PMN cell influx, relative numbers of alveolar macrophages (AM) in BAL declined proportionally (Figure 3A,B), while their total numbers remained stable and unaffected by infection (Supplementary Materials Figure S5A,B). At 48 h p.i., loss of pulmonary AM numbers was detected in lungs (Supplementary Materials Figure S4B), while infection-triggered recruitment of monocytes was most evident in BAL (Supplementary Materials Figure S5B). In blood, monocyte proportions tended to increase at 24 h p.i. (Supplementary Materials Figure S6A). At 48 h p.i., steady state of *de novo* monocyte egress from bone marrow and recruitment to lungs had likely emerged (Supplementary Materials Figure S6B). Of greatest relevance to the envisaged therapeutic usage in patients, absolute cell counts and proportions of innate immune cells in BAL and lungs as acquired by FACS analysis were not affected by phage treatment, irrespective of whether the animal was infected with *A. baumannii* or sham-infected (Figure 3, Supplementary Materials Figures S4 and S5). Likewise, quantification and differentiation of blood leukocytes showed no changes after phage application (Supplementary Materials Figure S6). Even 48 h p.i., no effect of phage-treatment on populations of innate immune cells in BAL, lungs, and blood was detected.

Furthermore, to exclude the possibility of phage treatment-specific changes in the release of pro-inflammatory mediators, levels of different cytokines and chemokines were determined in BAL fluid and plasma. Yet again, there were no differences found in sham-infected groups treated with either buffer or phages. As expected, CCL2, CCL3, CXCL1, and IL-6 levels were elevated in BAL fluid following *A. baumannii* infection. Most notably, when compared to buffer-treated group, there was no phage treatment-specific increase in cytokine and chemokine release at 24 h p.i. (Table 1). Whereas at 48 h p.i., cytokine levels were still increased in buffer-treated animals, phage-treated mice showed slightly attenuated cytokine levels. Notably, CCL-2 even showed significantly lower levels in BAL fluid of infected phage-treated animals compared to buffer treatment (Table 2). Likewise, systemic concentrations of G-CSF were found reduced in *A. baumannii* infected phage-treated mice when compared to the buffer-treated counterpart, while levels of CCL2 and CXCL1 were not affected significantly (Supplementary Materials Tables S3 and S4). Taken together, there was no phage treatment-specific induction of innate immune cells and inflammatory mediators in either *A. baumannii* or sham-infected immunocompetent animals.

### 3.3. Phage Treatment Eliminated A. baumannii in Human Lung Tissue without Concomitantly Increasing Proinflammatory Cytokine Release

**Figure 4:**
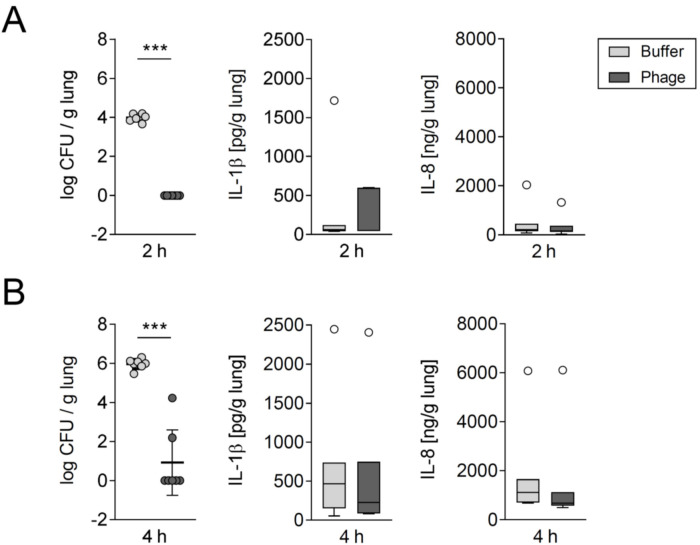
Phages lysed *A. baumannii* in human lung tissue without increasing proinflammatory cytokine release. Fresh human lung tissue was stimulated with 1 × 10^3^ CFU/mL *A. baumannii* RUH 2037. Thirty min after infection either phage vB_AbaM_Acibel004 or control solution (buffer) were injected. CFU and cytokine levels of IL-1β and IL-8 were determined 2 h (**A**) and 4 h (**B**) after treatment; *n* = 7, independent experiments. CFU data are expressed as single values with mean ± SD. CFU data were logarithmized (Y = log(CFU+1)) and analyzed with unpaired student’s *t*-test. *** *p* < 0.001 between indicated groups. Cytokine data are represented as box plots depicting median, quartiles and range excluding outliers (open circles) and analyzed using Mann-Whitney U Test.

Above stated findings in mice were validated in an ex vivo model using excised human lung tissues [34]. Accordingly, human lung tissues were stimulated with 1 × 10^3^ CFU/mL MDR *A. baumannii* and treated with a single-dose of 5 × 10^9^ PFU/mL vB_AbaM_Acibel004 phage or buffer 30 min later. Remarkably, no viable bacteria could be isolated after 2 h of phage treatment under the conditions tested (Figure 4A). After two additional h of phage treatment, bacterial viability remained compromised, except for 2 samples showing detectable growth (Figure 4B). As a representative of proinflammatory response markers, the release of IL-1β and IL-8 was measured in infected human lung tissue. However, there was no significant increase upon phage treatment at both time points (Figure 4A,B).

## 4. Discussion

Multidrug-resistant *A. baumannii* is of serious concern and local outbreaks at intensive care units are difficult to manage due to decreasing treatment options. Phage therapy might provide an effective resource in this regard. Our results show that a single application of a lytic bacteriophage reduced the viability of multidrug-resistant *A. baumannii* clinical isolate under ex vivo and in vivo conditions. Additionally, the treatment with phages controlled the progression of murine pneumonia without any further aggravation of inflammatory response and adverse off-target side effects. Thus, our data reaffirm the potential of phage therapy to treat MDR *A. baumannii* infections.

Using the preclinical model developed here, we were able to accurately analyze the clinical success of our phage candidate against *A. baumannii* infection. Given the generally low virulence of most *A. baumannii* isolates for healthy mice [3], induction of full-blown pneumonia is rarely successful unless additional tools are employed, such as immunosuppression or exogenous mucin application. Consistently, our results showed a self-limiting and nonlethal *A. baumannii* lung infection in immunocompetent mice. However, it allowed to assess the efficacy of phage treatment and its impact on a fully functional immune system. Another key aspect of our model is the avoidance of neutropenia, induced by cyclophosphamide in similar preclinical evaluation studies [38,39,40], which is an uncommon risk factor for *A. baumannii* infection in patients [3,41]. This aspect is crucial as recently dependence of phage therapy success on the synergistic relationship of phages with innate immune cells was established [42]. Along this line, our study methods such as initiating treatment 12 h p.i. in immunocompetent mice aimed to mimic a more clinically relevant setting and are unique as previous studies evaluated phage efficacy in immunosuppressed mice either after simultaneous application or short time periods (2–4 h) following *A. baumannii* infection [38,39,40]. Initiating phage treatment at a later timepoint allowed for an assessment of efficacy of delayed phage application, interaction with a more progressed immune response to bacterial challenge, and influence on established pulmonary vascular barrier failure.

Besides the optimal timing, the route of application is also expected to influence the success of treatment. In this study, we chose intratracheal aerosolization of phages in infected mice considering the potential clinical relevance, although the preferable route of application for lung infections is not yet clearly defined. Carmody et al. found that systemic application is superior over inhalation of phages in controlling *Burkholderia cenocepacia* lung infection in a murine model [43], whereas, Semler and colleagues showed the opposite [44], highlighting the difficulty in defining an ideal route of phage application. Nevertheless, inhaled application might be favorable for patients with pulmonary infections [45,46]. Advantages lie in the effective delivery of the active agent to the site of infection, the easy and comfortable use for the patient and avoidance of systemic phage exposure [47,48]. The latter is of special relevance because systemic application might increase the risk of producing antibodies against phages [49,50] or adverse immune responses including fever [51]. Moreover, phages are quickly cleared from the bloodstream when the density of the host bacterium is insufficient for phage propagation, impairing efficacy of systemically applied phages [52]. Regardless, studies concerning pharmacodynamics, pharmacokinetics, and tissue penetration of phages are needed to identify an appropriate route of application [45].

Single application of phages significantly reduced the bacterial viability, although unable to sterilize *A. baumannii* entirely from alveolar spaces and lung parenchyma in vivo, as expected due to phage amplification at the site of infection. This is consistent with results from other studies, showing the inability to kill entire bacterial population through single dose of intratracheally or intravenously applied phages [53,54,55,56]. Even adequate antibiotic therapy as well as combination therapy with phages or phage lysins does not necessarily kill all bacteria at once [30,54]. Most likely, we failed to reach the ideal multiplicity of infection for the phage-host system used in our study. Repeated intratracheal aerosolization of the phage solution was impossible in our model as it would have required repeated sedations within a short period of time and a high cumulative liquid load to murine lungs. Nose-only-inhalation systems may be considered as alternative approach. Future studies should address the possibility of recurrent and long-term use of purified phages, particularly regarding efficacy and possible humoral immune responses, such as the role of neutralizing antibodies to phages. Additionally, recent data point towards a potential benefit of combination therapy utilizing conventional antimicrobial drugs and phages. Sublethal concentrations of antibiotics were shown to augment phage efficacy [57,58,59], while on the other hand phage application can trigger changes in bacteria that reduce their virulence or even restore their susceptibility against antibiotics [60,61,62,63]. However, unfavorable antagonistic interactions between certain antibiotics and phages have also been described and thus need to be considered [64]. At present, general recommendations regarding combination of antibiotic and phage therapy cannot be given and need further investigation.

Critically ill patients with MDR *A. baumannii* infection require rapid treatment [3], thus time-consuming de novo search, isolation, characterization, and purification of an active phage is less favorable [65]. Accordingly, phage vB_AbaM_Acibel004 [21] was selected as the only appropriate lytic phage against the used clinical isolate of *A. baumannii* from an existing collection of nine well-characterized *A. baumannii* phages. In addition to rapid phage selection, a controlled and standardized production process of phage formulation with possibility of large scale production is a key prerequisite for the approval of bacteriophages as human therapeutics [66]. Importantly, endotoxins and other bacterial components that inherently remain in the solution during phage production could eventually elicit severe immune responses upon phage application [67]. Thus, our candidate phage was obtained after purification according to quality requirements for a pharmaceutical grade active ingredient [25]. So far, there are no guidelines defined for maximum endotoxin levels in formulations for inhalation therapy in humans. However, the European Pharmacopoeia specifies an endotoxin level of 5 EU/kg body weight per h for intravenous application which must be considered prior seeking approval of phages as therapeutics in the European Union [68]. We succeeded in substantially reducing the endotoxin levels in our phage preparation, yet it still exceeded the above-mentioned standard. It is encouraging, however, the residual endotoxin present in our phage preparation did not elicit any marked proinflammatory immune response. Nevertheless, any additional steps intended to remove such residual endotoxins might inadvertently affect the final phage yield and production costs. The preparation of purified phages therefore should be considered as important step during preclinical development of phages for inhalation therapy.

Furthermore, increased release of endotoxins by rapid phage lysis of target bacteria and the possible associated immune response are among the main reservations against phage therapy. When applied locally in the alveolar space, endotoxin-mediated damage to the pulmonary vascular barrier could further exacerbate edema formation in the infected lung [69]. As expected, *A. baumannii* infection was associated with lung barrier integrity failure in our mouse model, but phage-mediated lysis of the bacteria did not result in increased barrier failure and was even associated with its improvement. In line with our findings, lower lung edema formation and therefore improved lung barrier integrity after phage therapy was described by Dufour et al. comparing the influence of single intranasal phage application with antibiotic treatment in *Escherichia coli* (*E. coli*)-induced pneumonia [53]. Accordingly, the same authors demonstrated in different experiments with enteropathogenic *E. coli* that the amount of endotoxins released by phage lysis was comparable to the amount of endotoxins released by bactericidal antibiotics [70]. In our study, phage application per se did not result in any signs of barrier failure at all, as the air-blood barrier of sham-infected and phage-treated animals was unaffected by the phage solution.

Intratracheal phage aerosolization in our study was associated with reduced spread of bacteria in the lungs, presumably leading to a more local infiltration of immune cells and subsequently more concentrated areas of lung inflammation with reduced barrier breakdown, thus resulting in an improved clinical state of treated animals. In a previous study, Prazak et al. had shown an increase of IL-1β production in phage-treated uninfected animals [54], which was already reported by others [71], indicating a possible pro-inflammatory effect of the phage preparation itself. However, we failed to observe such phage effects neither in vivo nor in human lungs ex vivo.

Our study has some limitations. Since we used a clinical nonmouse-adapted *A. baumannii* isolate, our infection model was self-limiting and nonlethal but therefore allowed evaluation of the unimpaired innate immune response in immunocompetent mice. Future studies may address ventilator-associated pneumonia caused by MDR *A. baumannii* and infection of animals pretreated with antibiotics to mimic the clinical situation even better. In addition, finding lytic phages for *A. baumannii* turned out to be more difficult than for other species as Schooley et al. reported after treating a patient with *A. baumannii* sepsis as a last resort [13]. Therefore, our approach of using preselected well-characterized phages would be of great benefit for the application of adequate therapy to patients infected with MDR *A. baumannii*.

## 5. Conclusions

Our results showed the outcome of pneumonia following *A. baumannii* lung infection in immunocompetent mice could be improved through single dose of phage administration. Most importantly, the lack of inflammatory or other adverse effects in our study encourage further efforts to develop phages for clinical application against MDR *A. baumannii* pulmonary infections.

## Figures and Tables

**Table 1:** Levels of inflammatory mediators in BAL fluid 24 h p.i.

Inflammatory Mediator	Sham + Buffer (*n* = 8)	Sham + Phage (*n* = 8)	*A. baum.* + Buffer (*n* = 10)	*A. baum.* + Phage (*n* = 11)
CCL2	5.43 (11.70/5.43)	6.58 (9.73/5.72)	1859.06 (2381.62/533.71) #	778.07 (2586.93/220.98)
CCL3	1.75 (3.21/0.31)	1.25 (2.98/0.35)	1223.73 (2606.26/475.53) ##	445.54 (1415.85/80.79)
CXCL1	10.01 (18.72/4.14)	9.10 (16.04/4.67)	1948.90 (8826.17/1026.99) *p* = 0.0537	886.93 (2538.52/153.78)
IL-1β	0.81 (1.09/0.81)	0.81 (0.81/0.81)	47.32 (428.05/17.64)	16.89 (103.86/4.12)
IL-6	14.94 (62.06/5.72)	9.25 (44.25/3.99)	7804.57 (23,592.32/5289.06) #	2969.75 (14,636.74/1045.34)

Data are shown as median levels (interquartile range, 75th/25th percentile) of inflammatory mediators (pg/mL) and analyzed using 2-way ANOVA and Tukey’s multiple comparisons test; # indicates significant difference between infected vs. corresponding sham group; # *p* < 0.05 and ## *p* < 0.01; *A. baum., Acinetobacter baumannii*; CCL2, chemokine (C-C motif) ligand 2; CCL3, chemokine (C-C motif) ligand 3; CXCL1, chemokine (C-X-C-motif) ligand 1; IL-1β, Interleukin 1-β; IL-6, Interleukin 6, p.i., post infection.

**Table 2:** Levels of inflammatory mediators in BAL fluid 48 h p.i.

Inflammatory Mediator	Sham + Buffer (*n* = 8)	Sham + Phage (*n* = 8)	*A. baum.* + Buffer (*n* = 8)	*A. baum.* + Phage (*n* = 10)
CCL2	5.43 (6.29/4.77)	6.01 (6.58/5.43)	9772.22 (15525.95/4717.11) ###	2209.92 (4646.54/315.02) ***
CCL3	0.29 (0.66/0.29)	0.29 (0.52/0.24)	323.00 (428.44/296.65) #	263.22 (338.74/133.34)
CXCL1	4.14 (4.53/3.56)	4.14 (4.14/1.86)	521.75 (820.31/185.27) #	225.96 (361.35/51.88)
IL-1β	0.81 (0.81/0.81)	0.81 (0.81/0.81)	21.75 (67.19/16.79) #	21.66 (42.19/12.54) #
IL-6	4.86 (8.86/2.76)	2.17 (3.87/0.95)	4237.34 (6621.55/3279.73) ###	3698.67 (4663.81/2628.84) ###

Data are shown as median levels (interquartile range, 75th/25th percentile) of inflammatory mediators (pg/mL) and analyzed using 2-way ANOVA and Tukey’s multiple comparisons test; # indicates significant difference between infected vs. corresponding sham group; * *A. baumannii* + buffer vs. *A. baumannii* + phages; # *p* < 0.05 and ###/*** *p* < 0.001; *A. baum*., *Acinetobacter baumannii*; CCL2, chemokine (C-C motif) ligand 2; CCL3, chemokine (C-C motif) ligand 3; CXCL1, chemokine (C-X-C-motif) ligand 1; IL-1 β, Interleukin 1-β; IL-6, Interleukin 6; p.i., post infection.

## Data Availability

The complete susceptibility profile for *A. baumannii* RUH 2037 (DSM 101993) is publicly available at BacDive (https://bacdive.dsmz.de/strain/157916 doi:10.13145/bacdive157916.20201210.5, accessed on 29 November 2021).

## Supplementary Materials

The following are available online at https://www.mdpi.com/article/10.3390/v14010033/s1, Table S1: Drug susceptibility profile of *A. baumannii* RUH 2037. Table S2: Murine clinical disease scoring system. Table S3: Levels of inflammatory mediators in plasma 24 h p.i. Table S4: Levels of inflammatory mediators in plasma 48 h p.i. Figure S1: Innate immune cell gating strategy. Figure S2: Lysis of *Acinetobacter baumannii* by phage vB_AbaM_Acibel004. Figure S3: Relative body weight curves of mice. Figure S4: Phage treatment had no impact on innate leukocyte counts in the lungs. Figure S5: Phage treatment had no impact on innate leukocyte counts in BAL. Figure S6: Phage treatment had no impact on innate leukocyte counts in blood.

## Presentation of Results

The results presented here were reported in part as abstracts on scientific conferences: Herbsttagung der Sektionen Zellbiologie und Infektiologie und Tuberkulose der Deutschen Gesellschaft für Pneumologie und Beatmungsmedizin e.V., November 2017, Gießen, Germany, Georg Thieme Verlag KG; 2017; 59. Kongress der Deutschen Gesellschaft für Pneumologie und Beatmungsmedizin e.v., March 2018, Dresden, Germany, Pneumologie 2018; 72:S67-S68; European Respiratory Society International Congress, September 2018, Paris, France, Abstract No. PA5453, Respiratory infections, European Respiratory Society, 09152018:PA5453; American Thoracic Society International Conference, May, 2018, San Diego, California, USA, Abstract No. A7571; American Journal of Respiratory and Critical Care Medicine 2020;201:A7571.
