# Supplementary material for: Preclinical Assessment of Bacteriophage Therapy against Experimental Acinetobacter baumannii Lung Infection
Source: Viruses. 2021 Dec 24;14(1):33. doi: 10.3390/v14010033 (PMC8778864; doi:10.3390/v14010033)
Supplement: Supplementary file 1 [file viruses-14-00033-s001.zip › viruses-1514031-supplementary.pdf]

Preclinical assessment of bacteriophage therapy against experimental *Acinetobacter baumannii* lung infection

Sandra-Maria Wienhold*, Markus C. Brack*, Geraldine Nouailles, Gopinath Krishnamoorthy, Imke H. E. Korf, Claudius Seitz, Sarah Wienecke, Kristina Dietert, Corinne Gurtner, Olivia Kershaw, Achim D. Gruber, Anton Ross, Holger Ziehr, Manfred Rohde, Jens Neudecker, Jasmin Lienau, Norbert Suttorp, Stefan Hippenstiel, Andreas C. Hocke, Christine Rohde, Martin Witzenrath

* authors contributed equally

Supplemental Tables

Table S1. Drug susceptibility profile of *A. baumannii* RUH 2037

Antibiotic	Quantity per disc [µg]	Zone of inhibition [mm]
Penicillin G	6 (= 10 IU)	none
Oxacillin	5	none
Ampicillin	10	none
Ticarcillin	75	none
Mezlocillin	30	none
Cefalotin	30	none
Cefazolin	30	none
Cefotaxime	30	none
Aztrenoam	30	none
Imipenem	10	34-36
Tetracycline	30	none
Chloramphenicol	30	none
Gentamicin	10	6
Amikacin	30	20
Vancomycin	30	none
Erythromycin	15	10
Lincomycin	15	none
Ofloxacin	5	none
Norfloxacin	10	none
Colistin	10	14-16
Pipemidic acid	20	none
Nitrofurantoin	100	none

Antibiotic resistance profile was assessed via disk diffusion method according to DIN (Deutsches Institut für Normung e.V., German Institute for Standardization) standards. The complete susceptibility profile for A. baumannii RUH 2037 is publicly available at BacDive (https://bacdive.dsmz.de/strain/157916 doi:10.13145/bacdive157916.20201210.5).

Table S2. Murine clinical disease scoring system

Symptom	Score
**Fur**	
Shiny	0
Dishelved	1
**Eyes**	
Bright	0
Tearing	1
**Behavior**	
Curious, awaken	0
Self-isolation	1
Less active (cage-side observation)	1 or
Less reactive to external stimulus	2
**Breathing**	
Steady, consistent	0
Hyper- /hypoventilation	1 or
Labored	2
**Total clinical score**	

Table S3. Levels of inflammatory mediators in plasma 24 h p.i.

**Inflammatory mediator**	**Sham + Buffer (*n* = 8)**	**Sham + Phage (*n* = 8)**	***A. baum.* + Buffer (*n* = 10)**	***A. baum.* + Phage (*n* = 11)**
CCL2	18.71 (24.70/5.02)	21.28 (24.70/11.12)	91.24 (397.20/84.75)	101.74 (123.29/24.70)
CXCL1	2.62 (4.33/0.91)	4.33 (4.33/0.91)	71.39 (202.85/35.44)	19.90 (26.46/9.54)
G-CSF	8.16 (15.87/1.88)	6.76 (11.19/2.83)	588.15 (674.54/557.71) ###	351.25 (548.79/201.70) ###/***

Data are shown as median levels (interquartile range, 75th/25th percentile) of inflammatory mediators (pg/mL) and analyzed using 2-way ANOVA and Tukey´s multiple comparisons test; # indicates significant difference between infected vs. corresponding sham group, **A. baumannii* + buffer vs. *A. baumannii* + phages; ###/****p* < 0.001. *A. baum*., *Acinetobacter baumannii*; CCL2, chemokine (C-C motif) ligand 2; CXCL1, chemokine (C-X-C-motif) ligand 1; G-CSF, Granulocyte-colony stimulating factor; p.i., post infection.

Table S4. Levels of inflammatory mediators in plasma 48 h p.i.

**Inflammatory mediator**	**Sham + Buffer (*n* = 8)**	**Sham + Phage (*n* = 8)**	***A. baum.* + Buffer (*n* = 9)**	***A. baum.* + Phage (*n* = 10)**
CCL2	15.50 (24.70/6.29)	15.50 (24.70/3.16)	85.49 (130.24/51.21)	32.58 (70.64/24.70)
CXCL1	2.62 (4.33/0.91)	2.62 (4.33/0.91)	13.34 (59.31/5.23)	4.33 (6.79/4.22)
G-CSF	4.56 (7.94/1.48)	5.86 (8.18/2.73)	421.43 (625.27/254.27) ###	143.76 (192.42/61.94) ***

Data are shown as median levels (interquartile range, 75th/25th percentile) of inflammatory mediators (pg/mL) and analyzed using 2-way ANOVA and Tukey´s multiple comparisons test; # indicates significant difference between infected vs. corresponding sham group, **A. baumannii* + buffer vs. *A. baumannii* + phages; ###/****p* < 0.001. *A. baum*., *Acinetobacter baumannii*; CCL2, chemokine (C-C motif) ligand 2; CXCL1, chemokine (C-X-C-motif) ligand 1; G-CSF, Granulocyte-colony stimulating factor; p.i., post infection.

Supplemental Figures

Figure S1

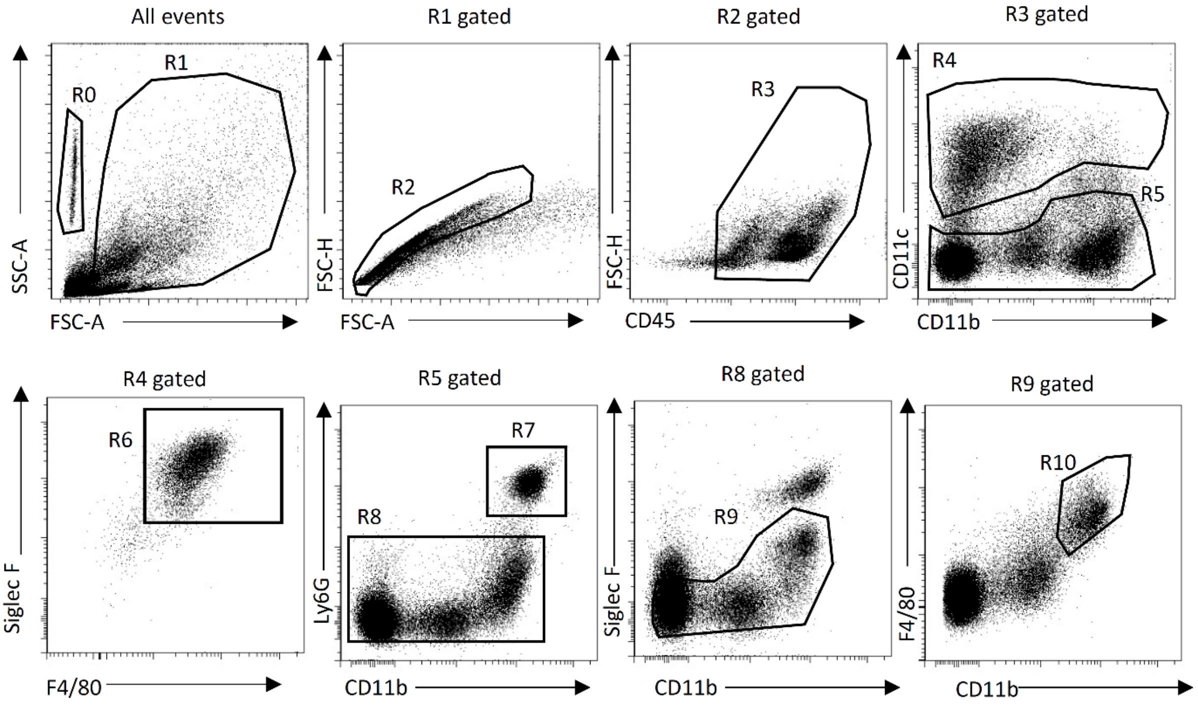

R0: Beads
R1: Minus debris
R2: Singlets
R3: CD45$^+$ **Leukocytes**
R4: CD11c$^+$
R5: CD11c$^-$
R6: CD11c$^+$Siglec F$^+$ F4/80$^+$ **AM**
R7: CD11c$^-$CD11b$^+$Ly6G$^+$ **PMN**
R8: CD11c$^-$Ly6G$^-$
R9: CD11c$^-$Ly6G$^-$Siglec F$^-$
R10: CD11c$^-$Ly6G$^-$Siglec F$^-$ F4/80$^+$CD11b$^+$ **iM**

Figure S1. Innate immune cell gating strategy. Dot blots indicate exemplary flow cytometric gating strategy of innate immune cell populations in alveolar spaces and lungs. AM, alveolar macrophages, PMN, polymorphonuclear leukocytes, iM, inflammatory macrophages.

Figure S2

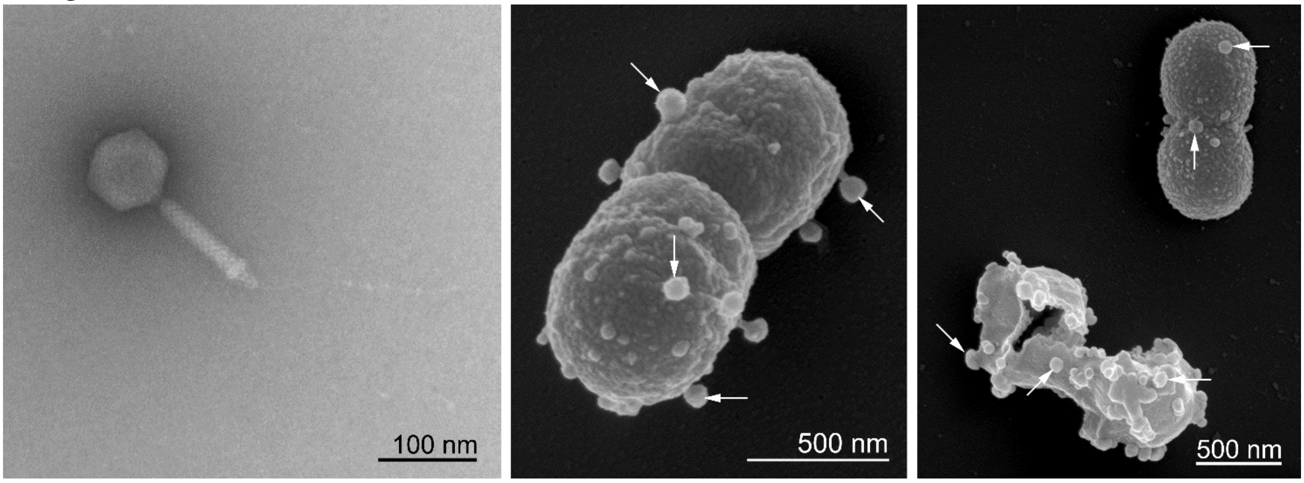

Figure S2. Lysis of *Acinetobacter baumannii* by phage vB_AbaM_Acibel004. Transmission electron micrograph (TEM) of negatively stained (2 % uranyl acetate) bacteriophage vB_AbaM_Acibel004 after diafiltration showing *Myoviridae* morphology with non-bended and contractile tail (left). Field emission scanning electron microscopy (FESEM) showing adherent phages (white arrows) to the surface of *A. baumannii* (middle) and showing remaining bacterial cell wall structures after lysis of *A. baumannii* RUH 2037 by phage vB_AbaM_Acibel004 (right picture, bottom). The top shows a bacterium with attached phages. Representative images out of 8 are shown.

Figure S3

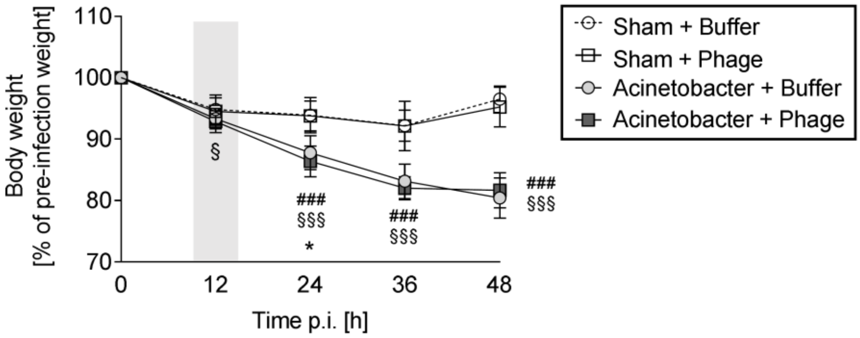

Figure S3. Relative body weight curves of mice. Mice were transnasally infected with *A. baumannii* RUH 2037 or sham-infected (DPBS) and treated intratracheally with a specific phage or control solution (buffer) 12 hours post infection (p.i.). Body weight was assessed twice daily for all mice. At defined analysis time points, mice were sacrificed. Relative body weight curves of all mice subjected to analysis at 24 h (*n* = 11-12; sham-infected groups or *n* = 16–17; *A. baumannii* infected groups) and 48 h (*n* = 12; sham-infected groups or *n* = 17; *A. baumannii* infected groups) are shown combined. Data are represented as mean ± SD and analyzed using 2-way ANOVA and Tukey´s multiple comparisons test; The grey box marks phage application time point; §/$^{\#}$/*indicate significant difference between groups at corresponding time point; $^{\#}$infected vs. corresponding sham group, *Acinetobacter + buffer vs. Acinetobacter + phages, §sham + phages vs. Acinetobacter + phages $^{*/\S}p < 0.05$ and $^{\#\#\#/\S\S\S}p < 0.001$.

Figure S4

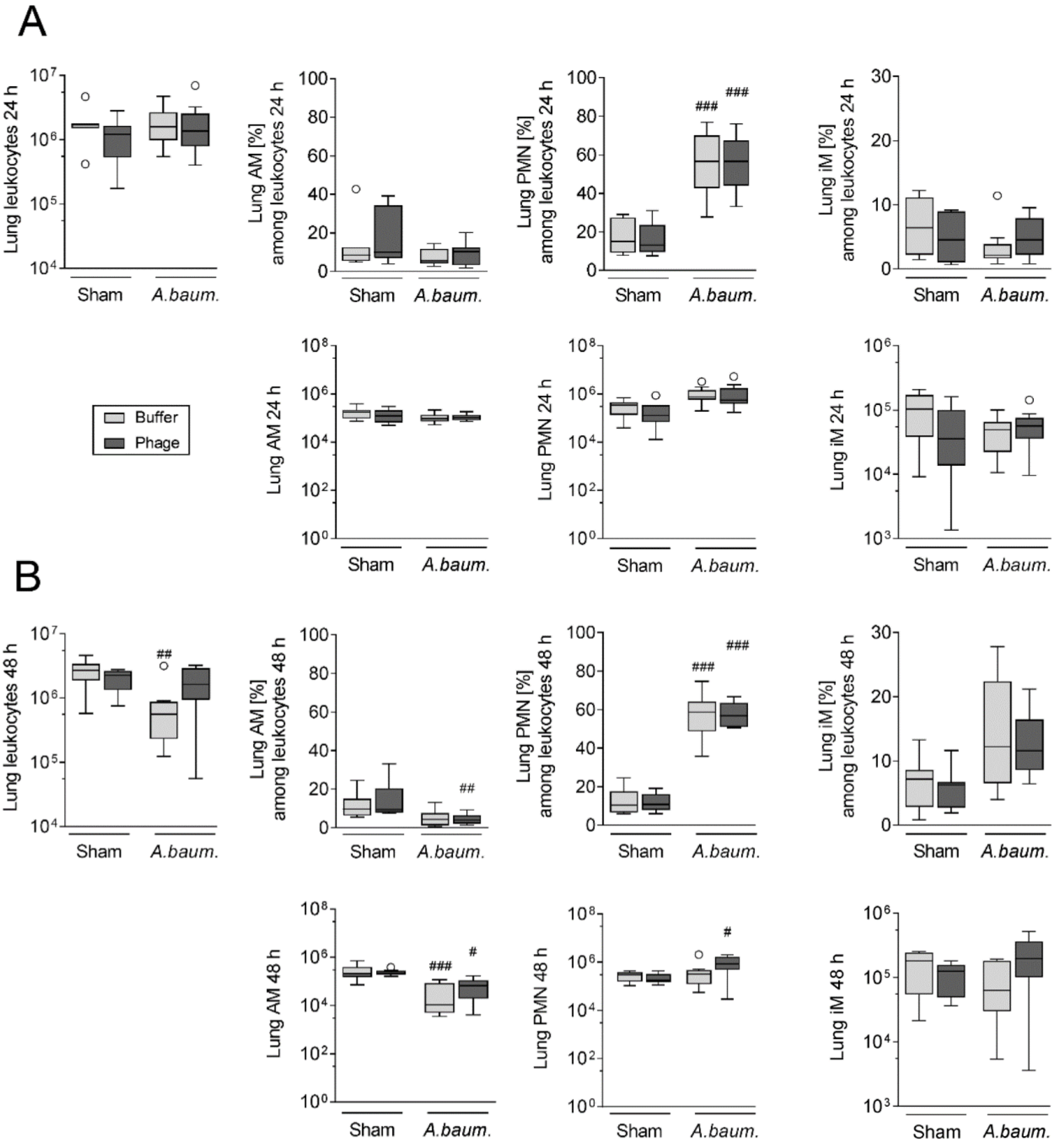

Figure S4. Phage treatment had no impact on innate leukocyte counts in the lungs. Numbers of leukocytes, alveolar macrophages (AM), inflammatory macrophages (iM) and polymorphonuclear leukocytes (PMN) in lavaged lungs were measured and percentages calculated 24 h (**A**) and 48 h post infection (p.i.) (**B**). Data are represented as box plots depicting median, quartiles and range excluding outliers (open circles) and analyzed using 2-way ANOVA and Tukey´s multiple comparisons test; n = 7-8 (sham infected groups); n = 9-11 (*A. baumannii* infected groups); $^{\#}$ indicates significant difference between infected vs. corresponding sham group, $^{\#}p < 0.05$, $^{\#\#}p < 0.01$, and $^{\#\#\#}p < 0.001$.

Figure S5

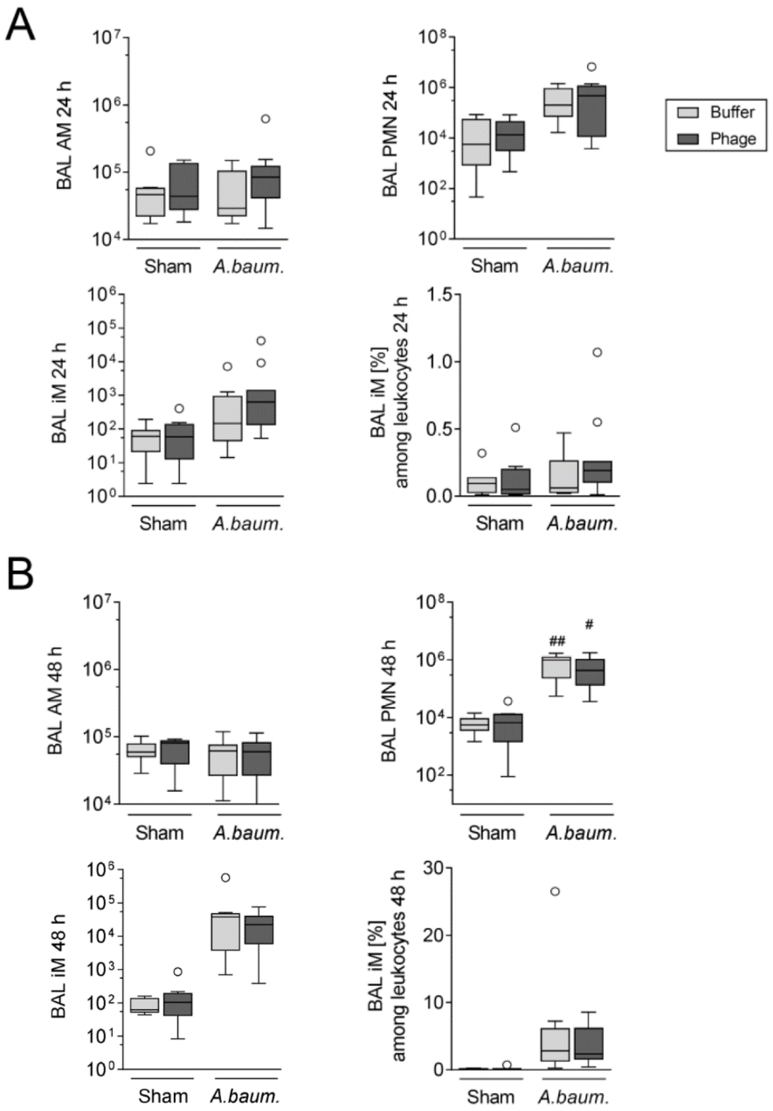

Figure S5. Phage treatment had no impact on innate leukocyte counts in BAL. Numbers of alveolar macrophages (AM), polymorphonuclear leukocytes (PMN), and inflammatory macrophages (iM) were measured in BAL and percentages of iM calculated 24 h (**A**) and 48 h post infection (p.i.) (**B**). Data are represented as box plots depicting median, quartiles and range excluding outliers (open circles) and analyzed using 2-way ANOVA and Tukey´s multiple comparisons test; *n* = 8 (sham infected groups); *n* = 8-11 (*A. baumannii* infected groups); # indicates significant difference between infected vs. corresponding sham group, # *p* < 0.05, ## *p* < 0.01

Figure S6

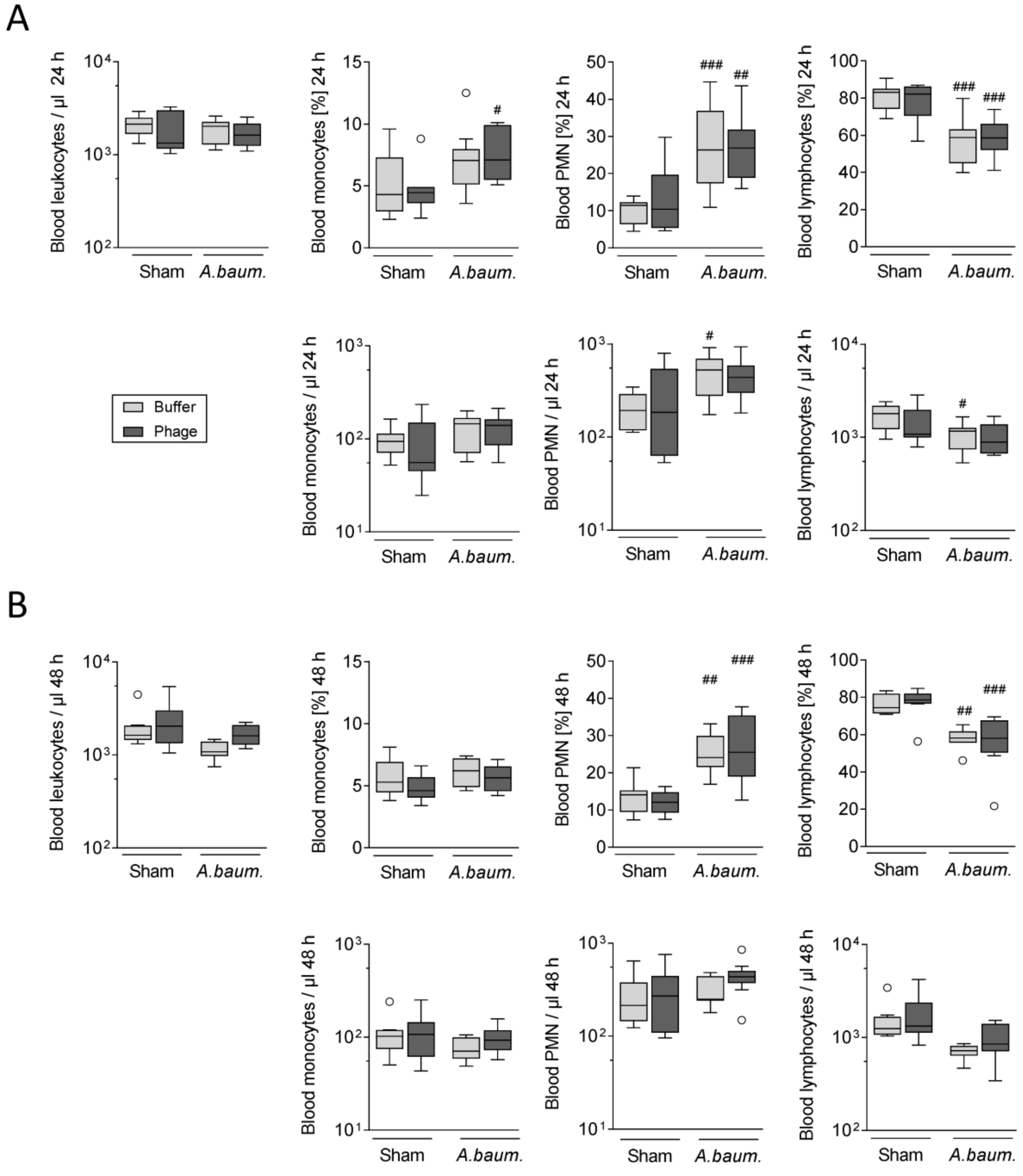

Figure S6. Phage treatment had no impact on innate leukocyte counts in blood. Numbers of leukocytes as well as numbers and percentages of monocytes, polymorphonuclear leukocytes (PMN), and lymphocytes in blood 24 h (**A**) and 48 h post infection (p.i.) (**B**) were measured in EDTA-blood by scil Vet abc hematology analysis. Data are represented as box plots depicting median, quartiles and range excluding outliers (open circles) and analyzed using 2-way ANOVA and Tukey´s multiple comparisons test; *n* = 8 (sham infected groups);

n = 7-11 (*A. baumannii* infected groups); # indicates significant difference between infected vs. corresponding sham group, $^{\#}p < 0.05$, $^{\#\#}p < 0.01$ and $^{\#\#\#}p < 0.001$.
